# Is GP-led follow-up feasible?

**DOI:** 10.1038/sj.bjc.6605667

**Published:** 2010-05-11

**Authors:** A B Francken, J W Hoekstra-Weebers, H J Hoekstra

**Affiliations:** 1 Division of Surgery, Isala Klinieken, Zwolle, The Netherlands; 2 Division of Surgical Oncology, University of Groningen and University Medical Centre Groningen, Groningen, The Netherlands; 3 Wenckebach Institute, University Medical Center Groningen, University of Groningen, Groningen, The Netherlands; 4 Comprehensive Cancer Center North East, Groningen, The Netherlands

During the last decennium, follow-up of cancer patients has been a major topic in oncology research. In melanoma, the need and benefit of follow-up has been discussed for many years and for several reasons. The incidence of melanoma is increasing, but without any increase in the mortality rate (Bataille and Vries, 2008). Consequently, the prevalence of melanoma is rising, with more melanoma patients in follow-up. Melanoma patients are reasonably young, and those who do not suffer from metastatic disease are often in a remarkably good condition because the initial melanoma treatment does not often dramatically impair patients’ physical functioning. Furthermore, the risk of recurrence is generally small (About 66% of melanoma cases at primary diagnosis are stage 1a or 1b and have a 10-year survival rate of more than 90 and 80%, respectively.) (Balch *et al*, 2009) and lastly, currently no definite therapeutic options are known once the metastatic disease presents.

The goal of melanoma follow-up is early detection of ‘curable’ disease, local recurrences, intransit metastases or nodal disease. Is an intensive melanoma follow-up schedule justified to reach this goal? Additional questions are (1) What do patients need in terms of follow-up?; (2) What kind of follow-up is sensible and can be offered?

Looking at the available literature, several aspects are of importance. No international consensus exists on how melanoma patients should be followed up after primary diagnosis, nor on the frequency of clinical visits, on the duration of follow-up or on which type of health-care professional to be consulted (Francken *et al*, 2005). There seems to be consensus only on omitting routine tests in follow-up, although some argue even on this aspect (Garbe *et al*, 2003; Nieweg and Kroon, 2006).

Follow-up is appreciated by patients (Francken *et al*, 2007). However, follow-up does not seem to have a role in the detection of recurrences. Many patients detect recurrences and second primary melanoma themselves (Francken *et al*, 2007, 2008). Self-examination seems to work well for many patients, but could be improved for optimal benefit (Boone *et al*, 2009). Upcoming therapies to treat patients with a recurrence effectively might change the follow-up concepts for melanoma patients in the near future. However, such therapies are currently available only in clinical trials (Seetharamu *et al*, 2009).

The article of Murchie *et al* (2010) presents a prospective randomised controlled trial on the feasibility of GP-led follow-up. Patients were randomised between a follow-up programme with their (recently trained) GP (intervention arm) and traditional follow-up at the hospital melanoma clinic at Aberdeen Royal Infirmary (control arm). Measurements were taken at baseline and follow-up done by considering patient satisfaction as the primary outcome and guideline adherence, quality of life, and anxiety and depression as secondary outcomes. They found higher patient satisfaction on several aspects of care received and better guideline adherence at follow-up in the intervention group than in the traditional hospital group, but not in quality of life or anxiety and depression. Consequently, their careful conclusion is that GP-led follow-up is feasible.

Several explanations can be given for the differences in satisfaction found between the groups. Although Murchie *et al* corrected for several biases (e.g., travel time to follow-up appointment), not all bias could be eliminated. Patients in the intervention group received a thorough skin examination by their GP and, in addition, were given an information booklet on how to conduct self-examination. This was not provided to the control group. For the purpose of the study, GPs had recently received a half-day training on how to examine and identify possible (recurrent) melanomas. Moreover, GPs in the intervention group had only a few melanoma patients to look after as compared with the hospital physicians. Which ingredient would have worked? Participation in the study, the recent training that the GPs had received, the information booklet on self-examination or the small number of patients that these GPs gave particular attention to? A longer study follow-up is needed to gain insight into patient satisfaction with GP follow-up once the melanoma follow-up is as routine as in the hospital. Satisfaction from the GPs may also change in the longer follow-up, because of increase in work pressure (Bakker *et al*, 2002).

Heterogeneity in this study was high. Patients with all types of melanoma ⩽4 mm Breslow thickness, as well as those with a time since primary diagnosis of 6 months up to 10 years, were eligible. These patients have a different prognosis and might have different levels of disease performance. Although the patients were randomised, heterogeneity might have influenced the outcome of the study.

The most important question is about safety for patients regarding morbidity, the detection of recurrences and mortality. This aspect could not be studied because thousands of patients would be needed, as explained by Murchie *et al.* We suspect that this would not be affected by GP follow-up because self-examination might anyway be the key to early detection.

In conclusion, the study by Murchie *et al* suggests that follow-up can be performed by GPs without affecting patient satisfaction. Follow-up of melanoma patients should be performed by well-informed/educated and dedicated people. Patients should get clear information on self-examination and their knowledge and performance should be regularly examined. Future research should focus on the contribution of providing this information to self-examination and the true effect on morbidity and mortality. Future studies may investigate the effect of regular examination of the skin by a nurse practitioner or by even the patients’ partner or significant other who has received a training comparable to the one the GPs had.

## References

[bib1] Balch CM, Gershenwald JE, Soong SJ, Thompson JF, Atkins MB, Byrd DR, Buzaid AC, Cochran AJ, Coit DG, Ding S, Eggermont AM, Flaherty KT, Gimotty PA, Kirkwood JM, McMasters KM, Mihm Jr MC, Morton DL, Ross MI, Sober AJ, Sondak VK (2009) Final version of 2009 melanoma staging and classification. J Clin Oncol 27: 6199–6206 19917835 10.1200/JCO.2009.23.4799 PMC2793035

[bib2] Bataille V, Vries E (2008) Melanoma – Part 1: epidemiology, risk factors and prevention. Br Med J 337: 1287–1291 10.1136/bmj.a2249 19022841

[bib3] Boone SL, Stapleton J, Turrisi R, Ortiz S, Robinson JK, Mallett KA (2009) Thoroughness of skin examination by melanoma patients: influence of age, sex and partner. Australas J Dermatol 50: 176–180 19659978 10.1111/j.1440-0960.2009.00533.x PMC2907135

[bib4] de Bakker DH, Hutten JB, Steultjens M, Schellevis F (2002) Rising workload or rising work pressure in general practice in the Netherlands. Eur J Publ Health 12: s48

[bib5] Francken AB, Bastiaannet E, Hoekstra HJ (2005) Follow-up in patients with localised primary cutaneous melanoma. Lancet Oncol 6: 608–621 16054572 10.1016/S1470-2045(05)70283-7

[bib6] Francken AB, Shaw HM, Accortt NA, Soong SJ, Hoekstra HJ, Thompson JF (2007) Detection of first relapse in cutaneous melanoma patients: implications for the formulation of evidence based guidelines. Ann Surg Oncol 14: 1924–1933 17357855 10.1245/s10434-007-9347-2

[bib7] Francken AB, Shaw HM, Thompson JF (2008) Detection of second primary melanomas. Eur J Surg Oncol 34: 587–592 17681449 10.1016/j.ejso.2007.06.004

[bib8] Garbe C, Paul A, Kohler-Späth H, Ellwanger U, Stroebel W, Schwarz M, Schlagenhauff B, Meier F, Schittek B, Blaheta HJ, Blum A, Rassner G (2003) Prospective evaluation of a follow-up schedule in cutaneous melanoma patients. J Clin Oncol 21: 520–529 12560444 10.1200/JCO.2003.01.091

[bib9] Murchie P, Nicolson MC, Hannaford PC, Raja EA, Lee AJ, Campbell NC (2010) Patient satisfaction with GP-led melanoma follow-up: a randomised controlled trial. Br J Cancer 102: 1447–1455 20461089 10.1038/sj.bjc.6605638 PMC2869159

[bib10] Nieweg OE, Kroon BB (2006) The conundrum of follow-up: should it be abandoned? Surg Oncol Clin N Am 15: 319–330 16632217 10.1016/j.soc.2005.12.005

[bib11] Seetharamu N, Ott PA, Pavlick AC (2009) Novel therapeutics for melanoma. Expert Rev Anticancer Ther 9: 839–849 19496721 10.1586/era.09.40

